# Multicentric Carpo-Tarsal Osteolysis Syndrome Mimicking Juvenile Idiopathic Arthritis: Two Case Reports and Review of the Literature

**DOI:** 10.3389/fped.2021.745812

**Published:** 2021-10-15

**Authors:** Junfeng Wu, Li Wang, Ye Xu, Zhiyong Zhang, Xin Yan, Yunfei An, Yu Zhang, Xuemei Tang

**Affiliations:** ^1^Department of Rheumatology and Immunology, Children's Hospital of Chongqing Medical University, Chongqing, China; ^2^National Clinical Research Center for Child Health and Disorders, Children's Hospital of Chongqing Medical University, Chongqing, China; ^3^Ministry of Education Key Laboratory of Child Development and Disorders, Children's Hospital of Chongqing Medical University, Chongqing, China; ^4^Chongqing Key Laboratory of Child Infection and Immunity, Children's Hospital of Chongqing Medical University, Chongqing, China; ^5^Chongqing Key Laboratory of Pediatrics, Children's Hospital of Chongqing Medical University, Chongqing, China; ^6^Department of Radiology, Children's Hospital of Chongqing Medical University, Chongqing, China

**Keywords:** multicentric carpo-tarsal osteolysis syndrome, MAFB protein, juvenile idiopathic arthritis, genetic testing, Denosumab

## Abstract

Multicentric carpo-tarsal osteolysis syndrome (MCTO) is a rare skeletal disorder commonly caused by MAF bZIP transcription factor B (*MAFB*) mutation. Clinically, it is characterized by aggressive osteolysis, which mainly affects the carpal tarsal bones, and is frequently associated with progressive nephropathy. Since the painful swelling and motion limitation on the wrists and/or ankles of MCTO mimics those of juvenile idiopathic arthritis (JIA), very often, MCTO is misdiagnosed as JIA. Here, we report two MCTO patients initially diagnosed with JIA but showed no response to treatment: P1, with a medical history of more than 10 years, was presented with a typical triad of arthritis-osteolysis-nephropathy; while P2 showed oligoarthritis. Gene tests were then taken and revealed a novel mutation, p.P63Q, and a previously reported conversion, p.S54L, in the *MAFB* gene. We also summarized the clinical and genetic features of a cohort containing 49 genetically confirmed MCTO patients. All 51 gene-confirmed MCTO cases (49 identified from the literature plus two patients identified herein) developed the disease during childhood. The median delay in diagnosis was 3.83 years (0–35 years). All cases presented bony lesions, and two-thirds had secondary renal lesions (32/48; three unknown), half of which (16/32) progressed into renal failure. Almost two-thirds (34/51), 75% (38/51), and 71% (36/51) of patients had no record of eye problems, facial abnormalities, and other manifestations. Most were misdiagnosed as JIA but didn't respond to treatment. Based on our experience, we suggest that clinicians should comprehensively evaluate the involvement of multiple systems in JIA patients, especially the kidney and eyes. And for JIA patients who underwent more than 3-month treatment with Bio-DMARD, genetic tests are recommended when they show little/no clinical and imaging changes, their high disease activity remains, and their disease activity remission is <50%, especially when combined with a triad of arthritis-osteolysis-nephropathy.

## Introduction

Multicentric carpo-tarsal osteolysis syndrome (MCTO, OMIM#166300) is a rare skeletal disorder characterized by early childhood onset of aggressive osteolysis, which significantly affects the carpal and tarsal bones, as well as other large joints such as the elbow and knee joints (1). Patients with MCTO often develop progressive nephropathy, leading to renal failure; they might also display subtle facial features, including a triangular shape, micrognathia, and exophthalmos (1). Most cases are sporadic, although familial cases have been reported.

MCTO is often misdiagnosed as juvenile idiopathic arthritis (JIA), especially as oligoarthritis because the painful swelling and motion limitation on the wrists and/or ankles of MCTO mimics those of JIA. However, they can be differentiated by clinical, laboratory, radiological, and genetic criteria (2). In contrast to JIA, MCTO has other clinical manifestations such as renal, ocular, and facial abnormalities. Apart from the triad of arthritis-osteolysis-nephropathy, joint involvement in MCTO patients usually begins from the wrist or ankle, which is rarely seen in oligoarticular JIA. In MCTO, reduction and eventually absence of pain, as well as the relief of wrist motions restriction is striking as time goes on. Typical osteolysis changes and complete bone loss can be found in MCTO patients, although laboratory investigations do not show signs of inflammation. In addition, MCTO patients can develop into family clusters.

Here, we report two Chinese boys of similar age but showing entirely different characteristics regarding the onset and progression of MCTO. Both were misdiagnosed with JIA and responded poorly to JIA treatment. Genetic test eventually confirmed the diagnosis of MCTO. We also reviewed the genetic and clinical manifestations of a larger cohort of MCTO patients reported in the literature to better understand this disease and help clinicians diagnose earlier, facilitating the treatment and management of the disorder.

## Case Reports

Patient 1 was a 12-year-old boy born to healthy unrelated parents with no family history of any skeletal lesions or nephropathy. His first rheumatologic symptoms (a painful, swollen right wrist, and limited passive and active flexion and extension of the right wrist) appeared at 2 years and 4 months old. His right 2^nd^-5^th^ proximal interphalangeal joints (PIPs) gradually became swollen and painful 6 months later. After excluding other known conditions, he was suspected of having oligoarticular JIA since <4 joints were involved during the first 6 months according to International League of Associations for Rheumatology (ILAR) classification criteria (3), and treated for 1 year with leflunomide (LEF) and diclofenac sodium. Although treatment relieved the swelling and pain in the wrist, significant restriction on the joint motion remained. Clinicians at another hospital added methotrexate (MTX) to the protocol, which continued for 3 years. Until he grew up to 7 years old, when he recovered from joint symptoms, his parents quitted the treatment concerning the side effects of the medication.

Between the ages of 7 and 11, the joint swelling or pain was absent, and no imaging examination or urine tests were performed. However, at 11 years old, his right elbow, right wrist, and right 2^nd^-5^th^ PIPs showed mild swelling and became painful over a few days to a week. A few months later, a stiff right elbow, with limited range of motion (both active and passive) and a flexion deformity, was noted. Magnetic resonance imaging (MRI) of the elbows was performed at 11 years and 8 months old, which showed an abnormal patchy signal in the right humeral olecranon fossa, joint cavity effusion, and synovial thickening. No X-ray of the wrists or other joints was taken because they were asymptomatic. He restarted JIA treatment with MTX, naproxen, and a tumor necrosis factor-α (TNFα) receptor inhibitor (etanercept, given for 3 months). However, there was no improvement in the lesions or subjective symptoms. A second MRI of the right elbow and wrist 7 months later revealed progression of the previous bone lesion, with joint deformation, damage to the articular surface (cartilage and bone), and synovitis. The carpal bones in the right wrist were missing. The left hand was not evaluated. Treatment was changed to MTX, sulfasalazine (SSZ), and diclofenac sodium for another 3 months, but with no symptomatic improvement.

**Figure 1:**
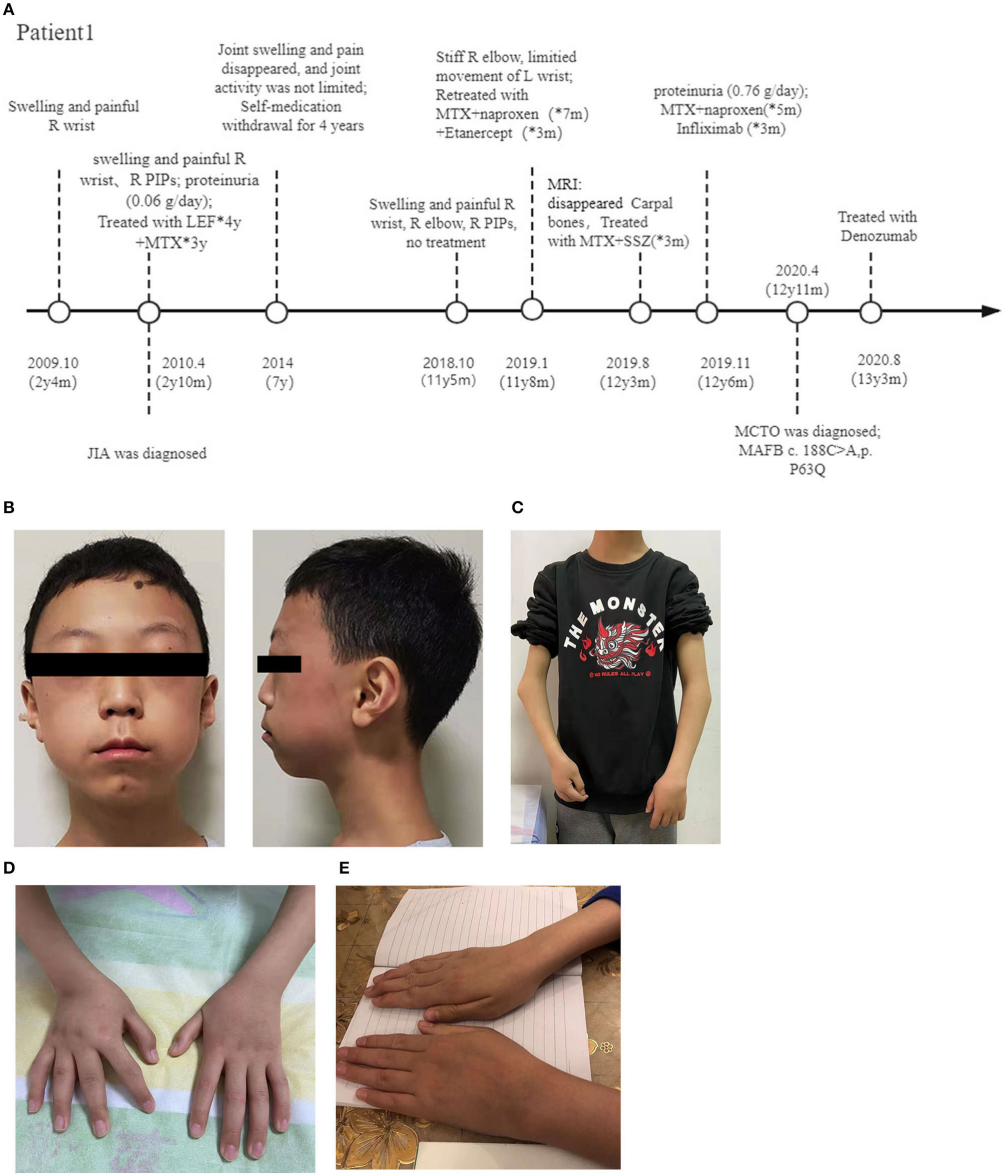
Photographs of the patients. **(A)** Diagram of the disease progression of patient 1. **(B)** Anteroposterior view of patient 1 at 12 years old, showing a triangular face, micrognathia, and a protruding forehead. View of the same patient showing elbow deformities and a short forearm **(C)**, and ulnar deviation of both wrists **(D)**. **(E)** Swollen left wrist of patient 2. R, right; y, year; m, month; PIP, proximal interphalangeal; MTX, Methotrexate; LEF, Leflunomide; SSZ, Sulfasalazine; MCTO, Multicentric carpo-tarsal osteolysis syndrome; MAFB, MAF bZIP transcription factor B.

The patient visited our hospital at 12.5 years old (Figure 1A). Although his parents were of average height, that of the patient was between the 10–25^th^ percentile (150 cm), and his weight was below the 3^rd^ percentile (30 kg). Physical examination revealed subtle facial abnormalities (protruding forehead and micrognathia), but no clinical evidence of an abnormality in the temporomandibular joints (Figure 1B). Respiratory, cardiovascular, and abdominal examinations were normal, and his cognitive function was appropriate for his age. He had marked angular malformation of the right elbow and ulnar deviation of both hands, which was more pronounced on the right (Figures 1C,D). Both wrist joints and bilateral 1^st^-5^th^ PIPs were swollen and painful, but his shoulders, hips, knees, and spine were not affected.

**Figure 2:**
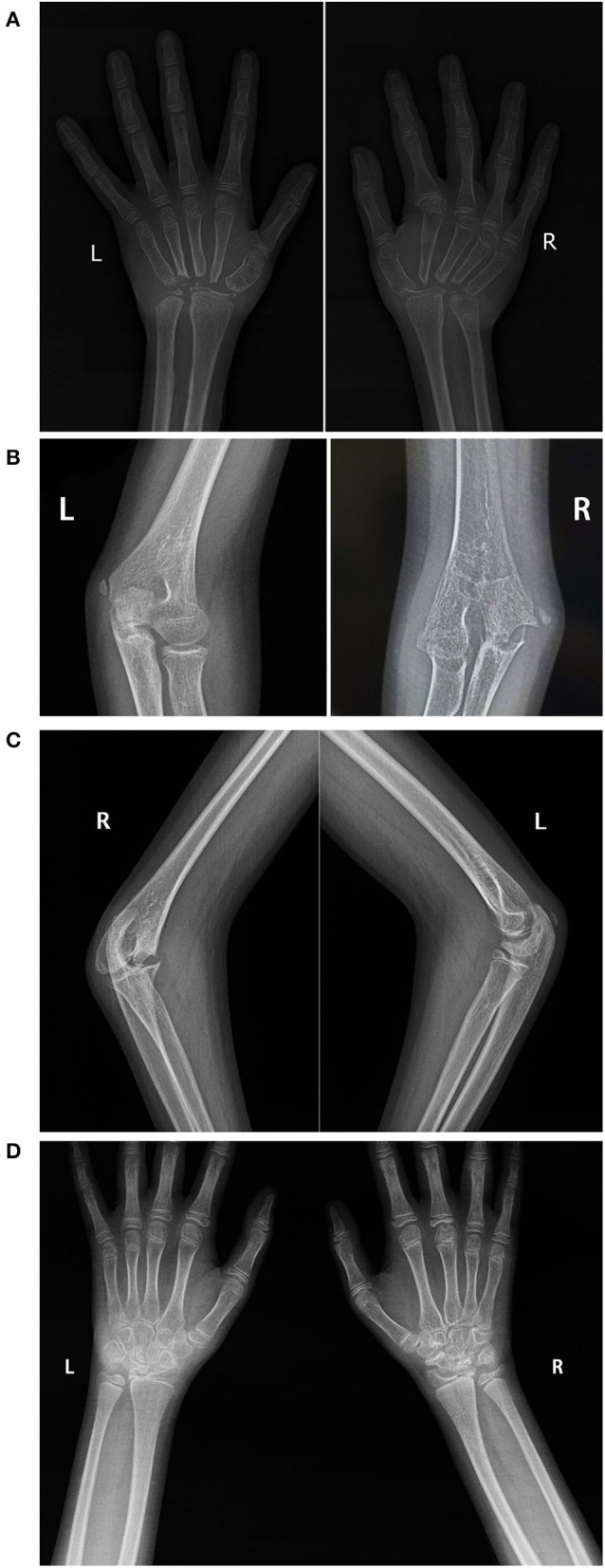
Radiological findings. X-ray of the hands of patient 1 **(A)**, taken at 12 years old, showing severe erosion of the carpal bones and the proximal metacarpal bones. X-ray of the elbows of patient 1 **(B,C)**, showing deformation of the distal right humerus and proximal ulnar and radius; destruction of the ossification center, narrowing of the space in the right elbow joint, and swelling of the soft tissue around the right elbow joint. X-ray of the wrist of patient 2 **(D)** showing abnormal bilateral carpal bone morphology, especially the proximal row of carpal bones.

Laboratory tests revealed normal antinuclear antibody, rheumatoid factor (RF), anti-citrullinated protein antibody levels, erythrocyte sedimentation rate (ESR) and C-reactive protein (CRP); elevated IL-6 (482.59 pg/ml, normal range: 0–16.6pg/ml) and TNF-α (12.45 pg/ml, normal range: 0–5.2pg/ml). Radiographs of the hands (Figure 2A) revealed osteoporosis and absence of most of the carpal bones; all PIPs and the metacarpophalangeal joints of the right hand were narrowed to some degree, and the second metacarpophalangeal joints of the right hand were deformed. The proximal part of the bilateral metacarpal was tapering. X-ray of the elbows (Figures 2B,C) revealed deformation of the distal right humerus and proximal ulnar and radius. Destruction of the ossification center, narrowing of the right elbow joint space, and swelling of the soft tissue around the right elbow joint was noted. The lower extremities were not affected, radiographs of both feet were normal (not shown).

Renal involvement began at 2 years old, when proteinuria (0.06 g/day), with normal renal function and normal blood pressure, was noted. The proteinuria resolved without treatment over the next 4 years of follow-up. During hospitalization (at the age of 12.5 years), proteinuria (0.76 g/day) was detected incidentally during a routine checkup; therefore, to assess renal involvement, we examined blood pressure, serum creatinine, albumin, and total cholesterol levels, all of which were normal. Renal ultrasound revealed compression of the left renal vein. Captopril was used to reduce urinary protein levels.

After being discharged from the hospital, he was treated as extended oligoarthritis, and his disease activity was assessed by clinical Juvenile Arthritis Disease Activity Score (cJDAS) 27 monthly, which was constantly high (identified by cJDAS > 4) (4). Even after 3 months of TNFα receptor inhibitor (infliximab) treatment, fewsignificant changes in clinical manifestations and imaging were seen, and his disease activity remission was <50% (cJDAS = 32 and 30, before and after infliximab treatment). Therefore, we considered his treatment was invalid and suggested he do a genetic test. The trio whole-exome sequencing (WES) results revealed a *de novo* heterozygous missense mutation at NM_005461: c.188 C > A (NP_005452.2: p. P63G) (Supplementary Figure 1A, Table 1). Comparative genomics analysis revealed conservation of proline 63 in the transactivation domain of MAF bZIP transcription factor B (*MAFB*) (Supplementary Figures 1C,D). According to American College of Medical Genetics and Genomics (ACMG) guidelines (5), this mutation is pathogenic (the revised ACMG criteria include PS2 + PM1 + PM2 + PM5 + PP3). The gene test results coupled with the typical clinical manifestations finally led to a diagnosis of MCTO. He was then treated with Denosumab (a single dose of 60 mg) at 13 years and 3 months old; 1 month later, his joint pain almost disappeared. He then received another two doses of Denosumab (60 mg per month, every 2 months). At the last follow-up (aged 13 years and 7 months) he had developed proteinuria (1.0 g/day), but renal function was normal. Swelling and pain in all affected joints were relieved significantly, and the rate of joint destruction had slowed (as assessed by MRI).

**Table 1:** Clinical and genetic characteristics of the two MCTO patients.

**Characteristics**	**Patient 1**	**Patient 2**
Gender	Male	Male
Age at onset of bone lesions	2 years 4 months	11 years and 9 months
Age at MCTO diagnosis	12 years	12 years and 3 months
Diagnostic delay	10 years	6 months
Age at onset of renal lesions	2 years	-
Age at onset of renal failure	–	–
Family history	–	–
Bone lesions	+	+
Joints (except wrists and ankles) affected	+	–
Renal lesions	+	–
Renal failure	–	–
Eye problems	–	–
Facial abnormality	+	–
Other manifestations	–	–
Treatment
NSAIDs	+	+
DMARDs	MTX, LEF, SSZ	MTX
TNFα inhibitors	Etanercept, Infliximab	Infliximab, Adalimumab
Denosumab	+	–
ACEI	+	–
Calcium supplements	+	+
*MAFB* variants	c.188 C > A, p. P63G	c.161C > T, p. S54L
Inherited derivation	*de novo*	*de novo*

*MCTO, Multicentric carpo-tarsal osteolysis syndrome; NSAIDs, Non-steroidal anti-inflammatory drugs; DMARDs, Disease-modifying antirheumatic drugs; MAFB, MAF bZIP transcription factor B; MTX, Methotrexate; LEF, Leflunomide; SSZ, Sulfasalazine; TNF α, Tumor necrosis factor-α; ACEI, Angiotensin-Converting Enzyme Inhibitors*.

Patient 2, a previously healthy male, developed swelling and pain in both wrists, along with limited movement at 11 years and 9 months old. 1 month later, he was hospitalized. Physical examination revealed that his height was in the 50–75^th^ percentile (155 cm), and his weight was in the 10–25^th^ percentile (35 kg). He had no facial dysmorphisms, and examination of his heart, lungs, abdomen, and neurological system was unremarkable. Swelling and tenderness were present in the wrist joints, particularly the left wrist (Figure 1E), and dorsiflexion of the 4^th^ metacarpophalangeal joints of both hands was limited.

Radiography of the wrists revealed abnormal morphology of the carpal bones on both sides, especially the proximal carpal bones. Destruction of the joints in the right wrist was more severe than that in the left (Figure 2D). Laboratory tests including ESR, CRP level, RF, and anti-citrullinated protein antibody levels, HLA-B27 status, urine tests, and renal function were normal. Bone mineral density tests suggested a reduction in bone mass. He was diagnosed with oligoarticular JIA and treated with naproxen, MTX, and calcium. However, there was no symptomatic improvement, so he received a 3-month course of the TNFα receptor inhibitor adalimumab at 11 years and 11 months old. But it did not work either. Therefore, a genetic test was suggested. Meanwhile, he received two doses of the TNFα receptor inhibitor (infliximab), whereupon the pain, and swelling in the wrists were relieved, but the range of motion (active and passive) in the wrist joints worsened.

The gene test results revealed a *de novo* heterozygous mutation, NM_005461:c.161C > T (NP_005452.2: p. S54L), in *MAFB* (Supplementary Figure 1B, Table 1). This mutation was in the highly conserved transactivation domain (Supplementary Figures 1C,D), which is reported to be pathogenic. And it confirmed a diagnosis of MCTO. At the last follow-up, he was aged 12 years and 5 months, and treatment with Denosumab was under consideration.

## Discussion

MCTO is a rare skeletal disease. Although it has been described over the years, definitive diagnosis and proper management remain a challenge. In this study, we reported two MCTO cases previously misdiagnosed as JIA and responded poorly to JIA treatment. And eventually, we identified them as MCTO caused by *MAFB* mutation using high-throughput sequencing.

Here, we also reviewed 49 cases of MCTO harboring specific genetic mutations in *MAFB* (6–21) since 2012, when Zankl et al. (6) first reported that a mutation in *MAFB* was responsible for MCTO. The *MAFB* mutations and clinical presentations are listed in Supplementary Tables 1, 2, together with the two new patients reported herein (making *n* = 51 patients in total). We also compared the major clinical features of the Chinese patients with other cohorts (Table 2).

**Table 2:** Comparison of the major clinical features between Chinese patients with those in other cohorts.

**Characteristics**	**Total**	***n***	**Chinese**	***n***	**Western**	***n***
	**(*n* = 51)**		**(*n* = 5)**		**(*n* = 46)**	
Gender ratio (male:female)	18:15	33	3:2	5	15:13	28
Age at onset of bone lesions (years)	2 (0–12)	29	2 (0.5–11.75)	5	2 (0–12)	24
Age at MCTO diagnosis (years)	11 (1.5–38)	31	12.1 (1.9–16)	5	9.25 (1.5–38)	26
Diagnostic delay (years)	3.83(0–35)	27	8 (0.35–10.7)	5	3.79 (0–35)	22
Age at onset of renal lesions (years)	5.83 (1.17–29)	21	5 (2–12)	3	6.415 (1.17–29)	18
Age at onset of renal failure (years)	14.5 (5–42)	12	10	1	17 (5–42)	11
Time to progress to renal failure (years)	3 (0–13)	10	5	1	1 (0–13)	9
Family history (%)	33 (16/49)	49	0 (0/5)	5	36 (16/44)	44
Bone lesions (%)	100 (51/51)	51	100 (5/5)	5	100 (46/46)	46
Joints except wrists and ankles affected (%)	97 (30/31)	31	80 (4/5)	5	100 (26/26)	26
Renal lesions (%)	67 (32/48)	48	60 (3/5)	5	67 (29/43)	43
Renal failure (%)	50 (16/32)	32	33 (1/3)	3	52 (15/29)	29
Eye problems (%)	29 (5/17)	17	0 (0/5)	5	42 (5/12)	12
Facial abnormality (%)	85 (11/13)	13	60 (3/5)	5	100 (8/8)	8
Other manifestations (%)	80 (12/15)	15	40 (2/5)	5	100 (10/10)	10

Most patients (46/51, 90%) were from countries other than China. Approximately one-third (16/49, two unknown) had a positive family history. The disease occurrence did not associate with gender since the male: female ratio was 18:15 (18 unknown). All patients developed the disease during childhood with a median onset age of bone lesions at 2 years (0–12 years). By contrast, the median diagnosis age of MCTO was 11 years (1.5–38 years), and the median diagnostic delay was 3.83 years (0–35 years). The median onset age of renal lesions was 5.83 years (1.17–29 years), while that of renal failure was 14.5 years (5–42 years), and the median time of progression from lesion identification to renal failure was 3 years (0–13 years).

Almost all patients have a delayed diagnosis, which varies from 2 months to 35 years. There are several reasons for the diagnostic delay: First, the previous diagnosis was based mainly on typical clinical manifestations such as osteolysis and renal involvement; however, it may take years for these to develop, which can lead to delay. Second, although the bony and renal lesions are the most prominent features, JIA patients with eye problems, facial abnormalities, and other manifestations are more likely to be suspected of having other diseases. However, nearly two-thirds (34/51), 74% (38/51), and 70% (36/51) of MCTO patients had no record of eye, facial, and other manifestations, suggesting a lack of awareness among clinicians about the disease. Ignorance of these clinical manifestations may prevent early diagnosis and treatment. Therefore, we suggest that clinicians comprehensively evaluate the involvement of multiple systems in patients with bony lesions, especially the kidney and eyes, and conduct long-term follow-up. Based on our experience, it is recommended to conduct genetic tests for JIA patients who underwent more than 3-month treatment with Bio-DMARD when they show little/no clinical and imaging changes, high disease activity remains, their disease activity remission is <50%, and especially when combined with a triad of arthritis-osteolysis-nephropathy.

MAFB, which is expressed widely by pancreatic α cells, renal podocytes, epidermal keratinocytes, hair follicles, and hematopoietic stem cells, functions during embryonic urethral formation (22–27). Thus, MAFB protein regulates various developmental processes, including osteoclastogenesis and renal development (23, 28), and possibly the development of other organs and systems. The clinical manifestations vary among patients.

All 51 patients had wrist/ankle involvement, although 97% (30/31, 20 unknown) reported involvement of other joints, mostly the elbow, knee, and hip joints, and four patients had scoliosis (Supplementary Table 2). Two-thirds (32/48, 3 unknown) of patients had renal lesions, and half of these (16/32) progressed into renal failure. Renal biopsies obtained from four patients with early renal involvement (biopsy was performed at 3, 4, 13, and 14 years old, respectively) revealed focal segmental glomerular sclerosis (FSGS) (10, 18, 19) (Supplementary Table 2). Overall, 29% (5/17) of patients had eye problems, with corneal opacity as the primary manifestation. Almost 85% (11/13) of probands had subtle craniofacial abnormalities, including triangular faces, micrognathia, maxillary hypoplasia, and consequent exophthalmos (22). In addition, 80% (12/15) of probands had other clinical manifestations, such as multiple organ and tissue involvement, including the respiratory system, the cardiovascular system, the nervous system, the circulatory system, the immune system, and the skin (Supplementary Table 1). However, these symptoms were usually sporadic and not observed repeatedly in our patients or other reported cases. Of note, nearly two-thirds (34/51), 75% (38/51), and 71% (36/51) of patients had no record of eye, facial, and other manifestations, respectively, which may have contributed to the delay in diagnosis.

There are differences with respect to the organs affected, the onset of disease, rate of disease progression, and disease severity. The mechanism underlying this heterogeneity is unknown. We were interested to find out whether the genotype may contribute to these differences. Therefore, we examined the relationship between gene mutations and clinical manifestations. All 51 cases harbored *MAFB* gene mutations, including two cases in which *MAFB* conversion was not described in detail (10, 16); thus, mutations in only 49 patients are listed in Supplementary Table 1. All mutations were missense and lay within a short region of the amino-terminal transcriptional activation domain (amino acids 54–71, see Supplementary Table 2, Supplementary Figure 2). We carefully compared the genotypes and clinical phenotypes of all patients but found no clear links. However, we found that the same *MAFB* mutation, p.S69L, may have varying clinical manifestations (14, 19). In addition, some patients with severe renal or skeletal changes may harbor mutations at different genetic loci. The relationship between genotype and phenotype needs more case observations and follow-up as well as in-depth study of the associated mechanism(s).

Currently, there are no recognized effective treatment options for bone lesions and nephropathy associated with MCTO. Treatment with NSAIDs, traditional disease-modifying antirheumatic drugs (DMARDs, e.g., MTX and LEF), glucocorticoids, and diphosphonates (e.g., alendronate and pamidronate) are ineffective against pain or osteolysis. Bio-DMARDs (e.g., etanercept, infliximab, adalimumab, tocilizumab, and abatacept) are not reported to slow down osteolysis progression appreciably, although etanercept, infliximab, and tocilizumab are reported to relieve pain (7, 16). Other drug treatments, such as calcium supplements, should be taken with caution since one patient developed a hypercalcemia-induced convulsive episode during treatment (21). For patients with severe bone damage, surgical treatment is necessary. Indeed, 12% (6/51) of patients underwent surgery for functional correction and it helped improve their day-to-day “function (7, 10, 17, 19). Of note, two patients treated with Denosumab, an anti-RANKL antibody showed fair improvement with not only less pain, but also a marginal slowdown in the rate of osteolysis in one patient, and partial improvement on the MRI 9 months later in the other patient (18, 29). RANKL-induced osteoclastogenesis is negatively regulated by MAFB, which is encoded by the *MAFB* gene, thus reducing MAFB expression can increase osteoclastogenesis (28). According to molecular pathogenesis and case reports, Denosumab might be the most promising drug to treat bony lesions. P1 received three doses of Denosumab (60 mg) over 4 months, and he seemed to respond well: joint swelling and pain, as well as inflammation, as assessed by MRI, were reduced. However, at present, there is no consensus about the dosage, the interval of use, or the mechanism of the action on Denosumab. Therefore, further studies are needed.

Regarding nephropathy, traditional oral steroids and/or other immunosuppressive drugs had no effect, although there was an exceptional case in which treatment with cyclosporine A was successful (30). For patients with proteinuria only, angiotensin-converting enzyme inhibitors (ACEI) can be used, although nearly half of patients progress into renal failure. Dialysis and kidney transplants will be needed.

To investigate whether patients of different ethnicities have different presentations, we compared Chinese patients' clinical manifestations and genetic information with those of other cohorts (Table 2). We found no differences in the gender ratio, onset age of bone and kidney lesions, and bone and renal involvement incidence. All Chinese MCTO patients were sporadic cases without a family history, and none had eye problems. In the observed cases, the proportion with kidney involvement, abnormal facial features, and other manifestations seems lower in Chinese patients than in non-Chinese patients. However, the age of MCTO diagnosis is higher, and the diagnostic delay is more prolonged in Chinese patients. Only one Chinese patient developed renal failure. Overall, there is insufficient data to say whether there are ethnic differences.

## Conclusions

MCTO is a rare skeletal disorder and is often misdiagnosed as JIA. Here, we report two newly diagnosed Chinese MCTO patients. The results emphasized the importance of genetic tests and systemic reviews for JIA patients who show insufficient responses to treatment.

## Data Availability Statement

The datasets presented in this article are not readily available due to patient ethical concerns. Requests to access the datasets should be directed to the corresponding author.

## Ethics Statement

The studies involving human participants were reviewed and approved by the Ethics Committee of Children's Hospital of Chongqing Medical University (2020-244-1). Written informed consent was obtained from the individual(s), and minor(s)' legal guardian/next of kin, for the publication of any potentially identifiable images or data included in this article.

## Author Contributions

JW: patient management, data collection, and manuscript writing. LW: manuscript review and data analysis. YX: data analysis. ZZ: manuscript review and interpretation. YA, XY, and YZ: patient management and data analysis. XT: manuscript review, data analysis, and interpretation. All the authors read and approved the final version of the manuscript.

## Conflict of Interest

The authors declare that the research was conducted in the absence of any commercial or financial relationships that could be construed as a potential conflict of interest.

## Publisher's Note

All claims expressed in this article are solely those of the authors and do not necessarily represent those of their affiliated organizations, or those of the publisher, the editors and the reviewers. Any product that may be evaluated in this article, or claim that may be made by its manufacturer, is not guaranteed or endorsed by the publisher.
