# Supplementary material for: Multicentric Carpo-Tarsal Osteolysis Syndrome Mimicking Juvenile Idiopathic Arthritis: Two Case Reports and Review of the Literature
Source: Front Pediatr. 2021 Oct 15;9:745812. doi: 10.3389/fped.2021.745812 (PMC8554157; doi:10.3389/fped.2021.745812)
Supplement: Supplementary file 1 [file Presentation_1.pdf]

Methods

Patients

This study was undertaken at Children's Hospital of Chongqing Medical University (CQMU). Both patients were admitted to hospital due to joint pain. After excluding infection and cancer, they were suspected of having JIA and were treated accordingly. However, they responded poorly. Therefore, DNA sequencing was carried out. The study was approved by the Ethics Committee of Children's Hospital of CQMU (2020-244-1) and complied with the tennets of the Declaration of Helsinki. Informed consent was obtained from both participants and their legal guardians.

Literature review

Since Zankl et al. first reported that a mutation in *MAFB* was responsible for MCTO in 2012, we searched for articles published between 2012 and January 2021 using the keywords "Multicentric carpo-tarsal osteolysis syndrome (MCTO)" or "MAF bZIP transcription factor B (MAFB)" in the PubMed, Google Scholar, Medline, Wanfang Med Online, and China National Knowledge Infrastructure (CNKI) databases. Only clear cases with a confirmed genetic diagnosis were reviewed and assessed as part of this study. In addition, references cited in important and significant articles were scanned. All hits from these sources were cross-checked.

Genetic analysis

DNA obtained from the two children, named patient 1 (P1) and patient 2 (P2), was subjected to whole-exome sequencing and targeted gene sequencing (MyGenostics, Beijing, China), as previously described(1,2). Mutations in the *MAFB* gene were verified by Sanger sequencing (MyGenostics) using the following primer pair targeting exon 1 of the *MAFB* gene: F (5'- GACGCTTGGTGATGATGGTG-3') and R (5'- CTCTTCTCCGCTCTTCCCC-3').

Supplementary Fig. S1. Pedigree and sequence chromatograms of both patients and their parents. (A) Patient 1 carries the *de novo* heterozygous mutation c.188 C>A (p. P63Q), and patient 2 (B) has the *de novo* heterozygous mutation c.161 C>T (p. S54L), in the *MAFB* gene. Both mutation loci are highly conserved (C, D).

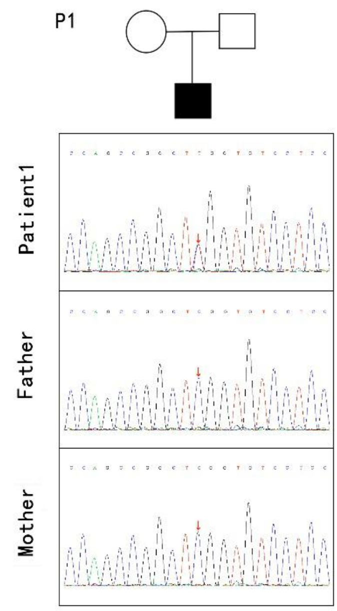

A

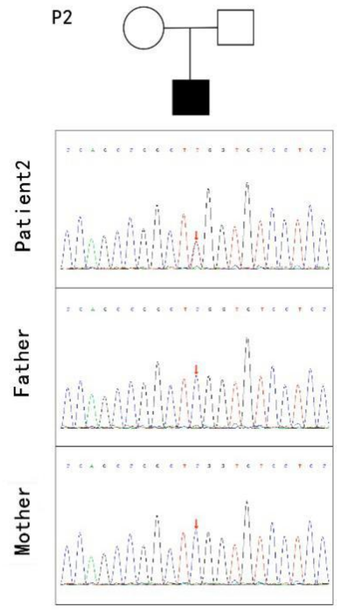

B

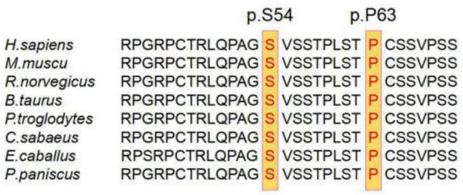

C

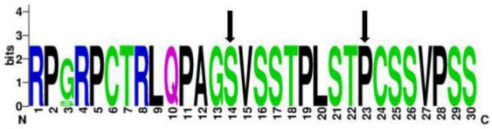

D

Supplementary Fig. S2 Mafb domain structure and the location of observed mutations in MCTO patients Each position indicates the amino acid substitution of the observed mutations in patients where mutations were previously reported for multicentric carpotarsal Osteolysis (MCTO). MCTO is known to be caused by a missense mutation in the transactivation domain. The red one indicates our patients' gene mutations. # (in blue) indicates sporadic cases, @ (in red) indicates familial cases.

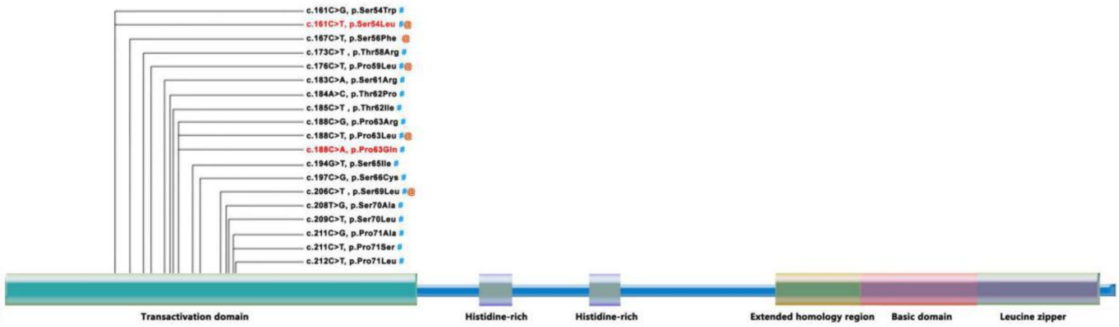

Supplementary Table S1. Summary of reported *MAFB* mutations in the MCTO patients

MAFB mutation		Probands		Reference
Nucleotide change	Amino acid change	Sporadic case	AD familial transmission	
161 C>G	Ser 54 Trp	1		(3)
161 C>T	Ser 54 Leu	2	7P/2F	(4,5), patient 2 in our study
167 C>T	Ser 56 Phe		1P/1F[a]	(6)
173 C>T	Thr 58 Arg	1		(2)
176 C>T	Pro 59 Leu	3[b]	2P/1F[c]	(3,4,7,8)
183 C>A	Ser 61 Arg	1		(9)
184 A>C	Thr 62 Pro	1		(4)
185 C>T	Thr 62 Ile	1		(7)
188 C>G	Pro 63 Arg	2		(3,4)
188 C>T	Pro 63 Leu	2	2P/1F	(3,10)
188 C>A	Pro 63 Gln	1		Patient 1 in our study
194 G>T	Ser 65 Ile	1		(3)
197 C>G	Ser 66 Cys	2		(4,11)
206 C>T	Ser 69 Leu	4	4P/2F[d]	(3,4,12,13)
208 T>G	Ser 70 Ala	1		(4)
209 C>T	Ser 70 Leu	3		(4,7)
211 C>G	Pro 71 Ala	1		(9)
211 C>T	Pro 71 Ser	3		(4,7)
212 C>T	Pro 71 Leu	3		(4,9,14)

a, The same *MAFB* mutation was found in the proband's unaffected mother, sister, and maternal grandmother; b, one of the proband's parents had no sample available for gene testing; c, the mother, who had clinical manifestations of MCTO, did not undergo gene testing; d, two patients were reported by both Mumm et al. (2014) and Regev et al. (2020).

P, patient; F, family.

Supplementary Table S2 Summary of the clinical characteristics of reported MCTO patients with *MAFB* mutations

Patients	Symptom onset(years)	Initial diagnosis (years)	Age at MCTO diagnosis (years)	Other Joints (except wrists and ankles) affected	Treatment and effectiveness	Renal findings	Eye problems	Facial abnormality	Other manifestations	Reference
P1-P11	NA	NA	NA	NA	NA	Five had undergone renal transplantation, three had markedly impaired renal function but did not yet require dialysis. Three had no evidence of renal dysfunction.	NA	NA	NA	(4)
P12-P14	NA	NA	NA	NA	NA	no evidence of renal dysfunction	NA	NA	NA	(4)
P15-P18	NA	NA	NA	NA	NA	no evidence of renal dysfunction, an exception of one individual who at a very old age developed renal dysfunction	NA	NA	NA	(4)
P19	R hand pain and swollen wrists(4y);	JIA	NA	NA	NA	NA	NA	NA	an Arnold-Chiari malformation type I. During late puberty, androgenetic alopecia and slight non-immunological bilateral exophthalmos	(6)
P20	wrists pain (2y)	JIA (2y)	17y	elbows, knees, hips,	salicylate(2y, no effective); Multiple orthopedic surgeries (childhood);	terminal renal insufficiency (17y), Peritoneal dialysis, and renal transplantation (8m later)	band keratitis with bilateral anterior stromal corneal opacities without	hypertelorism, exophthalmia, synophrys, hypoplastic alienate, protruding columella, and	Diaphragmatic hernia and underwent surgery (at birth); recurrent ENT complications (5y); EBV+ B cell	(3)

						corneal thinning	a chin dimple.	lymphoma with (28y)	
P21 (son)	left ankle pain (1y)	NA	11y	elbows, knees, hips, and thoracic scoliosis(18y)	orthopedic surgeries (15-16y); thoracic vertebral arthrodesis (18y); etanercept (5y-20y, pain release)	mild proteinuria (11y) and regular follow-up of proteinuria; enalapril (15y) +losartan(16y)	NA	NA	NA	(3)
P22 (Father of P21)	NA	NA	20y	elbows, knees	NA	renal insufficiency, renal transplantation (20y)	NA	NA	NA	(3)
P23 (son)	asymptom, but skeletal radiographs showed carpal osteolysis(5y10m)	NA	6y	phalanges of thumbs and forefingers	NA	HUS, bilateral renal hypoplasia (5y10m), dialysis, renal transplantation (6.5y)	NA	NA	several bronchiolitis and asthma in child hood;	(3)
P24 (Mom of P23)	carpal and tarsal osteolysis(2y)	Haidu Cheney syndrome	2y	distal forearms	None	high BP and proteinuria, but normal renal function (during first pregnancy at 29y), and symptoms resolved after delivery, preeclampsia with high BP and severe proteinuria, normal renal function (during 2nd pregnancy, 35y); acute renal insufficiency and bilateral renal hypoplasia(42y); Dialysis was	NA	NA	NA	(3)

						needed, waiting for renal transplantation				
P25	left ankle arthritis(3y)	Septic arthritis (3y); Torg Winchester syndrome (7y)	NA	left knee (10y), left elbow (11y)	surgical management (3y), casts and NSAIDs (10y); etanercept 12y (no effective, withdraw in 6m), physiotherapy (13y)	no renal impairment	NA	hypertelorism, high nasal bridge, and synophrys	NA	(3)
P26	wrist and foot abnormalities (early childhood)	NA	early childhood	NA	NA	terminal renal insufficiency (17y), Peritoneal dialysis was used until kidney transplantation (18y)	NA	NA	epileptic seizures from age 4 to 12 years, and was treated by valproate	(3)
P27	swollen wrists (6m)	NA	2y	right ulnar	NA	Normal renal function	NA	NA	NA	(3)
P28	Birth	JIA 2.5y	3y	shoulders, knees, elbows	NA	proteinuria (3 y)	corneal clouding	NA	NA	(7)
P29	6m	JIA	1.5y	elbows	NA	proteinuria (4 y), renal transplant （11y）	corneal clouding	NA	NA	(7)
P30	2m		1.5y	elbows, knees	NA	proteinuria (21 m), unsuccessful renal transplant （9 y）	none	NA	NA	(7)
P31	2m	Rickets	3y	TMJ, shoulders, knees, elbows	NA	proteinuria (5 y)	none	NA	NA	(7)
P32	2.5y	JIA	14y	elbows	NA	proteinuria (7 y),renal transplant （17 y）	none	NA	NA	(7)
P33	NA	JIA 6y	11y	elbows	NA	proteinuria (14y)	none	NA	NA	(7)
P34	12m	JIA	6.5y	elbows	NA	none	corneal clouding	NA	NA	(7)

P35	20m for symptoms	JIA (5 y)	5.5y	elbows, knees	Denosumab (less pain and increased daily activities with improved R wrist function, osteolysis stabilized)	none	none	NA	NA	(7,13)
P36 (Mom of P35)	3y	JIA	38y	cervical spine, elbows, PIP joints of Hands	NA	renal failure, shrunk kidney	none	NA	NA	(7,13)
P37	claudication symptoms (26m)	JIA	15y	feet and pes cavus.	NSAIDs, MTX(no effective)；infliximab (5y, pain decreased and eventually disappeared, progressive osteolysis); tocilizumab (8y, after three months treatment, the pain and tenderness disappeared)	NA	NA	micrognathia, hypotelorism, chubby cheeks, and flat face.	NA	(15)
P38	wrists, elbows, and shoulders pain and scoliosis (8y)	JIA	16y	elbows, shoulders, hips, and severe thoracic scoliosis	decompression and spinal fusion(12y), Combined Arthritis Program (CAP,16y), Total Hip Arthroplasty (16y)	bilateral renal cysts, and non-nephrotic range proteinuria (12y)	NA	mid-face hypoplasia, exophthalmos, micrognathia,	severe Chiari I malformation with herniation; mild dilatation of the aortic root and a restrictive pattern on pulmonary function tests without parenchymal lung disease; mild central obstructive sleep apnea	(8)
P39	R wrist pain (4y)	NA	13y	ulna and radius	intra-articular injections of glucocorticoids, methotrexate(4 y); etanercept (7y)(progressive Bone destruction) ；	Concomitant proteinuria and kidney biopsy (13y), which revealed low-grade focal glomerulosclerosis	NA	NA	NA	(5)

					denosumab (18y, reduce the inflammation)					
P40	6m	NA	14y	elbows, all fingers, knees, feet	NA	Ultrasonography showed a small size of both kidneys, end-stage renal disease ((oliguria, anemia, and failure to thrive, 12y); dialysis and medications (erythropoietin, ferrous fumarate, calcitriol, and elemental calcium)	cloudy cornea, exophthalmos,	underdeveloped alanasi, maxillary hypoplasia, and micrognathia	Echocardiography demonstrated left ventricular hypertrophy; tonic-clonic seizure from hypercalcemia (12y)	(11)
P41	wrists and ankles (toddler)	NA		elbow, right femoral epiphysis	NA	asymmetry in the size of the kidneys (2y)； significant kidney hypoplasia of the right kidney, the left kidney was compensatorily hypertrophic, 13y); normal renal function	NA	triangular face, eye-bulging, micrognathia	olecranon bursitis, marfanoid habitus, cachexia, cutis laxa	(10)
P42	deformity of the left foot and pain in the right wrist (2y)	JIA (2y)		mandible and elbows, knees	prednisolone, methotrexate, and ibuprofen (no effective); alendronate (9y, no effective)	proteinuria(4y), oral steroid and enalapril, and proteinuria maintained; kidney biopsy revealed FSGS, NOS variant; chronic hemodialysis (5y), kidney transplantation (6y)	NA	Not mention	cleft palate，did corrective surgery (3y)	(9)

P43	deformity of the right thumb (12y)	JIA (12y)	14y	multiple PIP joints	correctional surgery, ibuprofen (14y, No effective)	isolated 2+ proteinuria (12y), A kidney biopsy revealed FSGS, NOS variant (14y); enalapril.	NA	Not mention	not mention	(9)
P44	tenderness hands and feet (3m)	NA		elbow, and knees	NA	A kidney ultrasonogram revealed diffusely increased renal parenchymal echogenicity, proteinuria (14m); normal renal function with spontaneous remission of proteinuria (22m), normal (4y)	NA	NA	an abdominal aortic aneurysm (14m)	(9)
P45	wrists pain and the bottom of his right foot with morning stiffness	ANA+ pJIA (6y)	7.5y	feet, right temporomandibular joint, and PIP joints of fingers	5y: MTX, etanercept, infliximab, abatacept, and tocilizumab, pamidronate (progressive arthritis); adalimumab	None	None	prominent forehead, maxillary hypoplasia, and bilateral palpebral ptosis, as well as a long philtrum and bulbous nose	speech and walking delay, Learning disability, Hearing impairment	(12)
P46 (Mom of P45)	wrists and elbows pain (5y)	erosive arthritis (late childhood)	33y	elbows, feet	NA	NA	NA	wide-set eyes, triangular face, small and thick ears, hypoplastic nares, a bulbous tip of the nose, and micrognathia	Learning disability	(12)
P47	NA	NA	7y	hands, elbows, and scoliosis	surgical treatment for scoliosis.	Diagnosed with FSGS due to proteinuria (3y) proteinuria, no renal	NA	a small forehead and hypotelorism;	NA	(16)

						dysfunction or hypertension				
P48	multiple bone deformities and an inability to walk (1.5y)	NA	10y		Bone biopsy (5y);	1+ proteinuria without hematuria (5y); deteriorated renal function (10y); Shrunk kidney, hemodialysis (10y), kidney transplant (11y)	None	triangular face, micrognathia, and exophthalmos	None	(2)
P49	Elbow and knee flexion (6m)	None	1.9y	Elbows, knees, and feet	Alfacalcidol	None	None	None	Mental Retardation	(14)
P50	Right Wrist pain (2y)	JIA (2y)	12.7y	Elbows	NSAIDs, DMARDs (LEF, MTX), and Pavlin (2-7y , joint pain and swelling were relieved and disappeared); naproxen, diclofenac sodium, NSAIDs, MTX, SSZ, etanercept, infliximab (11-13y, not practical); Denosumab (13y, joint pain released)	proteinuria	None	subtle facial abnormalities of protruding forehead and micrognathia	None	Patient1 in our study
P51	Wrists pain (11y9m)	JIA (11y10m)	12.1y	multiple proximal interphalangeal joints	naproxen , MTX, calcium, adalimumab, not effective	None	None	None	None	Patient 2 in our study

P: patient; JIA: Juvenile idiopathic arthritis; MCTO: Multicentric carpo-tarsal osteolysis syndrome; NA: not available; R: right; LEF : leflunomide; MTX: Methotrexate; SSZ: Sulfasalazine; FSGS: Focal Segmental Glomerular Sclerosis; NOS: not otherwise specified; TNF α: Tumor Necrosis Factor α; NSAIDs: nonsteroidal anti-inflammatory drugs; DMARDs: disease modifying antirheumatic drugs; CAP: Combined Arthritis Program; PIP: proximal interphalangeal

References

1. Jiang J, Tang W, An Y, Tang M, Wu J, Qin T, Zhao X. Molecular and immunological characterization of DNA ligase IV deficiency. *Clin Immunol* (2016) **163**:75–83. doi:10.1016/j.clim.2015.12.016
2. Li J, Shi L, Lau K, Ma Y, Jia S, Gao X. Identification of a novel mutation in the MAFB gene in a pediatric patient with multicentric carpotarsal osteolysis syndrome using next-generation sequencing. *Eur J Med Genet* (2020) **63**:103902. doi:10.1016/j.ejmg.2020.103902
3. Mehawej C, Courcet JB, Baujat G, Mouy R, Gérard M, Landru I, Gosselin M, Koehrer P, Mousson C, Breton S, et al. The identification of MAFB mutations in eight patients with multicentric carpo-tarsal osteolysis supports genetic homogeneity but clinical variability. *Am J Med Genet Part A* (2013) **161**:3023–3029. doi:10.1002/ajmg.a.36151
4. Zankl A, Duncan EL, Leo PJ, Clark GR, Glazov EA, Addor MC, Herlin T, Kim CA, Leheup BP, McGill J, et al. Multicentric carpotarsal osteolysis is caused by mutations clustering in the amino-terminal transcriptional activation domain of MAFB. *Am J Hum Genet* (2012) **90**:494–501. doi:10.1016/j.ajhg.2012.01.003
5. Zhuang L, Adler S, Aeberli D, Villiger PM, Trueb B. Identification of a MAFB mutation in a patient with multicentric carpotarsal osteolysis. *Swiss Med Wkly* (2017) **147**:w14529. doi:10.4414/smw.2017.14529
6. Dworschak GC, Draaken M, Hilger A, Born M, Reutter H, Ludwig M. An incompletely penetrant novel MAFB (p.Ser56Phe) variant in autosomal dominant multicentric carpotarsal osteolysis syndrome. *Int J Mol Med* (2013) **32**:174–178. doi:10.3892/ijmm.2013.1373
7. Mumm S, Huskey M, Duan S, Wenkert D, Madson KL, Gottesman GS, Nenninger AR, Laxer RM, McAlister WH, Whyte MP. Multicentric carpotarsal osteolysis syndrome is caused by only a few domain-specific mutations in MAFB , a negative regulator of RANKL-induced osteoclastogenesis. *Am J Med Genet Part A* (2014) **164**:2287–2293. doi:10.1002/ajmg.a.36641
8. Sun K, Barlow B, Malik F, Inglis A, Figgie M, Goodman S. Total Hip Arthroplasty in a Patient with Multicentric Carpotarsal Osteolysis: a Case Report. *HSS J* ® (2016) **12**:177–181. doi:10.1007/s11420-015-9478-0
9. Park PG, Kim KH, Hyun HS, Lee CH, Park JS, Kie JH, Choi YH, Moon KC, Cheong H Il. Three cases of multicentric carpotarsal osteolysis syndrome: A case series. *BMC Med Genet* (2018) **19**:1–7. doi:10.1186/s12881-018-0682-x
10. Stajkovska A, Mehandziska S, Stavrevska M, Jakovleva K, Nikchevska N, Mitrev Z, Kungulovski I, Zafiroski G, Tasic V, Kungulovski G. Trio clinical exome sequencing in a patient with multicentric carpotarsal osteolysis syndrome: First case report in the Balkans. *Front Genet* (2018) **9**:1–6. doi:10.3389/fgene.2018.00113
11. Choochuen P, Rojneuangnit K, Khetkham T, Khositseth S. The First Report of Multicentric Carpotarsal Osteolysis Syndrome Caused by MAFB Mutation in Asian. *Case Rep Med* (2018) **2018**: doi:10.1155/2018/6783957
12. Upadia J, Gomes A, Weiser P, Descartes M. A Familial Case of Multicentric Carpotarsal Osteolysis Syndrome and Treatment Outcome. *J Pediatr Genet* (2018) **07**:174–179. doi:10.1055/s-0038-1657760
13. Regev R, Laxer R, Whitney-Mahoney K, Elia Y, Noone D, Shammas A, Reza V, Sochett EB. MON-LB72 Multicentric Carpotarsal Osteolysis Syndrome (MCTO) Has a Generalized High Turnover Bone Phenotype, High S RANKL and Responds to Denosumab. *J Endocr Soc* (2020) **4**:771–772. doi:10.1210/jendso/bvaa046.2142
14. Zhi-kuan G, , ZHAI Hong-yin, ZHANG Yong TP. Multicentric carpotarsal osteolysis syndrome: a case report GUO. *CHIN J Osteoporos BONE Min RES Vol* (2019) **12**:8–12. doi:10 . 3969 /j.issn.1674-2591 . 2019 . 06 . 008
15. Nishikomori R, Kawai T, Toshiyuki K, Oda H, Yasumi T, Izawa K, Ohara O, Heike T. Remarkable improvement of articular pain by biologics in a Multicentric carpotarsal osteolysis patient with a mutation of MAFB gene. *Pediatr Rheumatol* (2015) **13**:P152. doi:10.1186/1546-0096-13-s1-p152
16. Miyazaki K, Komatsubara S, Uno K, Fujihara R, Yamamoto T. A CARE-compliant article: A case report of scoliosis complicated with multicentric carpotarsal osteolysis. *Medicine (Baltimore)* (2019) **98**:e17828. doi:10.1097/MD.0000000000017828
